# Marker-trait association analysis of functional gene markers for provitamin A levels across diverse tropical yellow maize inbred lines

**DOI:** 10.1186/1471-2229-13-227

**Published:** 2013-12-28

**Authors:** Girum Azmach, Melaku Gedil, Abebe Menkir, Charles Spillane

**Affiliations:** 1 International Institute of Tropical Agriculture, Oyo Road, Ibadan, PMB 5320, Nigeria; 2 Genetics & Biotechnology Laboratory, Plant and AgriBiosciences Research Centre, School of Natural Sciences, National University of Ireland Galway, Aras de Brun, Galway, Ireland

**Keywords:** Provitamin A, Carotenoids, Functional markers, Marker assisted selection, Biofortification, Vitamin A deficiency

## Abstract

**Background:**

Biofortification of staple crops is a cost effective and sustainable approach that can help combat vitamin A and other micronutrient deficiencies in developing countries. PCR -based DNA markers distinguishing alleles of three key genes of maize endosperm carotenoid biosynthesis (*PSY1*, *lcyE* and *crtRB1*) have been developed to facilitate maize provitamin A biofortification via marker assisted selection. Previous studies of these functional DNA markers revealed inconsistent effects. The germplasm previously employed for discovering and validating these functional markers was mainly of temperate origin containing low frequencies of the favourable allele of the most significant polymorphism, *crtRB1*-5′TE. Here, we investigate the vitamin A biofortification potential of these DNA markers in a germplasm panel of diverse tropical yellow maize inbred lines, with mixed genetic backgrounds of temperate and tropical germplasm to identify the most effective diagnostic markers for vitamin A biofortification.

**Results:**

The functional DNA markers c*rtRB1*-5′TE and *crtRB1*-3′TE were consistently and strongly associated with provitamin A content across the tropical maize inbred lines tested. The alleles detected by these two functional markers were in high linkage disequilibrium (R^2^ = 0.75) and occurred in relatively high frequency (18%). Genotypes combining the favourable alleles at the two loci (N = 20) displayed a 3.22 fold average increase in β-carotene content compared to those genotypes lacking the favourable alleles (N = 106). The *PSY1* markers were monomorphic across all of the inbred lines. The functional DNA markers for *lcyE* were associated with lutein, and with the ratio of carotenoids in the alpha and beta branches, but not with provitamin A levels. However, the combined effects of the two genes were stronger than their individual effects on all carotenoids.

**Conclusions:**

Tropical maize inbred lines harbouring the favourable alleles of the *crtRB1*-5′TE and 3′TE functional markers produce higher levels of provitamin A. Such maize lines can be used as donor parents to speed up the development of provitamin A biofortified tropical maize varieties adapted to growing conditions and consumer preferences, providing a route towards mitigation of vitamin A malnutrition in Sub-Saharan Africa.

## Background

Carotenoids are naturally occurring organic pigments produced by plants mainly as integral component of the light capturing and protective plastidal apparatus [1]. Carotenoid intake plays an important role in human nutrition and health owing to the association of their consumption levels with reduced risk of diseases such as cardiovascular disease, cancer, and age-related sight problems arising from deficiencies of lutein and zeaxantine [2,3]. Provitamin A carotenoids including α-carotene, β-carotene and β-cryptozanthine are precursors of vitamin A, a micronutrient essential for normal development and functioning of the human body [4,5]. Vitamin A deficiency is currently a global public health problem inflicting morbidity, stunted growth, night blindness and loss of both sight and lives in the developing world [6,7]. Vitamin A deficiency is estimated to affect 190 million preschool children and 19 million pregnant and/or lactating women world wide [8], which aggravates poverty and underdevelopment challenges in developing countries [9,10].

Maize can naturally accumulate both provitamin A and non-provitamin A carotenoids in its kernel, and is known for its genetic diversity of carotenoid content and profiles [11-13]. However, provitamin A usually constitutes only 10 to 20% of the total carotenoids in maize kernel, and the commonly cultivated and consumed yellow maize cultivars have less than 2 μg g^-1^ DW provitamin A [14]. Exploitation of the natural genetic diversity of maize in carotenoids through biofortification by combining conventional and molecular breeding can increase provitamin A concentration in maize endosperm [15,16]. Such increases in provitamin A concentration can be beneficial for public health in Africa where maize is a major security and staple crop for more than 300 million people [17,18]. Biofortification offers a safe, effective, cheap and sustainable approach to combating vitamin A and other micronutrient deficiencies [14,19-23].

One of the major challenges in maize breeding for high provitamin A levels is the quantification of carotenoids in the endosperm of large number of breeding lines. High performance liquid chromatography (HPLC) is the commonly used method for carotenoids analysis because of its accuracy. However, HPLC is expensive, time consuming and relatively low throughput, limiting its use for routine breeding within resource-limited plant breeding programs [24]. Even though the variation in intensity of yellow color in maize endosperm is attributable to the variations in carotenoid content and profile, selection for high provitamin A maize based on kernel color is not reliable due to its poor correlations with provitamin A content [12,25]. Marker assisted selection [26] using functional DNA markers [27] offers an effective tool for screening a large number of breeding materials for their carotenoid profile and content accurately and cheaply within a reasonable timeframe within a plant breeding program.

The carotenoid biosynthesis pathway is well studied in plants [28-31], where the genes encoding the enzymes of the biosynthesis pathway are known [1,32]. Specific nucleotide sequence variants within the key carotenogenic genes have also been characterized, and shown to contribute significantly to accumulation of provitamin A and total carotenoids in maize endosperm [12,33,34]. For instance, [12] showed that four polymorphic sites in the gene encoding lycopene epsilon cyclase (*lcyE*) were associated with the variation in ratio of carotenoids in the α to β branches of the carotenoid biosynthesis pathway, leading to a threefold increase in provitamin A. [34] also identified three polymorphic sites in another downstream gene encoding a β-carotene hydroxylase enzyme (*crtRB1*) accounting for 40% of the observed variation in β-carotene concentration in maize endosperm. Two polymorphisms in the gene encoding phytoene synthase (*PSY1)* have been identified explaining 7 to 8% of the variation in total carotenoids [33]. These findings allowed the development of breeder friendly PCR based functional DNA markers that can be used as tools for detecting alleles representing each of the polymorphic sites in the three genes.

While these functional DNA markers can be used to facilitate the development of maize cultivars fortified with high provitamin A, their efficacy in breeding lines used for maize variety delivery to Sub-Saharan Africa has not been fully elucidated. Some studies have examined the individual and combined effects of the functional polymorphisms of *lcyE* and *crtRB1* on carotenoids [11,33,35,36]. It has been observed that the proposed diagnostic polymorphisms for *lcyE* could not distinguish between inbred lines with high lutein and high zeaxanthine, representing carotenoids in the α- and β-branches of the pathway, respectively [11]. Inbred lines having high β-carotene contents have been identified, although they were carrying the unfavourable alleles of *crtRB1*-5′TE and -3′TE. Similar inconsistencies for the favourable allele of the *crtRB1-*3′TE marker have been observed [36]. A recent study tested two of the three significant polymorphic sites of *lcyE* (5′TE and 3′ indel) and one of the three functional polymorphisms of *crtRB1* (3′TE) using 26 tropical segregating populations [35]. Their results showed that the effects of *lcyE* on both ratio of α to β branch carotenoids and total provitamin A content were inconsistent across the populations, whereas the *crtRB1-*3′TE polymorphic site had a large effect on β-carotene and provitamin A concentrations. In contrast, significant effects have been detected for all the functional polymorphisms for individual and haplotypes of selected polymorphisms of *lcyE, crtRB1* and *PSY1,* using inbred lines with tropical, subtropical and temperate backgrounds [33]. The reported inconsistencies in the effects of the diagnostic DNA markers for the proposed favourable alleles of *lcyE* and *crtRB1* require further investigation of these markers using diverse inbred lines to identify the most robust and effective markers for marker assisted selection.

In the present study, the functional DNA markers for *lcyE, crtRB1* and *PSY1* were tested on a set of diverse tropical inbred lines with mixed genetic backgrounds of tropical and temperate origin developed within the International Institute for Tropical Agriculture (IITA) maize breeding program for Africa. The inbred lines were first evaluated for carotenoid content and composition across two seasons (in 2010 and 2011) and exhibited contrasting variations. Two of the functional markers of *crtRB1* (i.e. the 5′TE and 3′TE markers) were found to be in high linkage disequilibrium, and displayed consistent and strong effect on the provitamin A carotenoid contents of the inbred lines. The deployment of these functional DNA markers can accelerate the biofortification of tropical-adapted maize varieties with elevated levels of provitamin A.

## Methods

### Plant materials

One hundred and thirty diverse tropical adapted yellow maize inbred lines were assayed for carotenoid profiles and content and used in this marker-trait association study. These inbred lines were developed within the maize breeding program for Africa at the International Institute for Tropical Agriculture (IITA) from eight bi-parental crosses of tropical inbred lines, four broad based populations, and 28 backcrosses involving temperate lines as donors of high β-carotene (Table 1).

**Table 1:** Maize genotypes used in present study

**Serial no.**	**Origin**	**Number of lines**
**1**	4001	1
**2**	9450	1
**3**	KU1409	1
**4**	9450/KI28	2
**5**	9450/KI21	6
**6**	9450/CM116/9450	2
**7**	9450/KI21-1-4-1-1-1-B/DE3/9450/KI21-1-4-1-1-1-B	4
**8**	9450/KI28-1-2-1-1-B/DE3/9450/KI28-1-2-1-1-B	3
**9**	9450/KI21-1-4-1-1-1-B/DE3/9450/KI21-1-4-1-1-1-B	1
**10**	9450/KI21-1-5-3-2-1-B/DE3/9450/KI21-1-5-3-2-1-B	1
**11**	9450/KI21-1-5-3-2-2-B/DE3/9450/KI21-1-5-3-2-2-B	2
**12**	9450/KI21-3-2-2-1-3/KU1409/MO17LPA/KU1409	1
**13**	DE3/KU1414-SR/KU1414-SR	3
**14**	KU1409/NC358/KU1409	3
**15**	KU1414-SR	1
**16**	KU1414-SR/KVI11	2
**17**	KU1409/DE3/KU1414-SR	1
**18**	KU1409/DE3/KU1409	23
**19**	KU1409/KU1414-SR/A619	9
**20**	KU1409/KU1414-SR/KVI11	1
**21**	KU1409/KU1414-SR/KVI3	12
**22**	KU1409/KU1414-SR/M162W	3
**23**	KU1409/KU1414-SR/NC298	8
**24**	KU1409/KU1414-SR/NC350	10
**25**	KU1409/KU1414-SR/SC55	2
**26**	KU1409/SC55/KU1409	4
**27**	KU1414-SR/CI7/KU1414-SR	2
**28**	KU1414-SR/CML328/KU1414-SR	1
**29**	POP66SR/ACR91SUWAN1-SRC1/ACR91SUWAN1-SRC1-1/SYN-Y-STR-34-1-1-1-1-2-1-B*3	2
**30**	POP66SR/ACR91SUWAN1-SRC1/ACR91SUWAN1-SRC1-4/4001/KI21-4-1-1-1-1	2
**31**	POP66SR/ACR91SUWAN1-SRC1/ACR91SUWAN1-SRC1-6/(MP420/4001/MP420)	3
**32**	POP66SR/ACR91SUWAN1-SRC1/ACR91SUWAN1-SRC1-8/POP61-SR-11-2-3-3-1-B	2
**33**	POP66SR/ACR91SUWAN1-SRC1/ACR91SUWAN1-SRC1-9/(9450/CM116/9450)-3-3-1-2-1	1
**34**	SYN-Y-STR-34-1-1-1-1-2-1-B*3/(DE3/CI7)/SYN-Y-STR-34-1-1-1-1-2-1-B	1
**35**	SYN-Y-STR-34-1-1-1-1-2-1-B*5/NC354/SYN-Y-STR-34-1-1-1-1-2-1-B*5	1
**36**	SYN-Y-STR	1
**37**	4205/CI7/4205	1
**38**	ACR97TZL-CCOMP1-Y-S3-13-1-B*2/CI7/ACR97TZL-CCOMP1-Y-S3-13-1-B*2	1
**39**	ACR97TZL-CCOMP1-Y	2
**40**	SC55/KU1414-SR/KU1414-SR	1
**41**	TZE-COMP5-Y-C7	1
**42**	Z.Diplo BC4	1

### Field evaluation

The 130 inbred maize lines were field evaluated at IITA’s research site (7°29′11.99″N, 3°54′2.88″E, altitude 190 m) in Ibadan, Nigeria, in 2010 and 2011. The field trial was arranged in a 13 × 10 alpha-lattice design with two replications. Each inbred line was planted in a single 5 m long row with spacing of 0.75 m between rows and 0.25 m between plants within a row. Different fields were used in each season. Fertilizer and field management practices recommended for optimum maize production were used. Seed samples for carotenoid analyses were produced by self pollination of at least 5 representative plants in each row. Self-pollinated ears in each row were harvested, dried under ambient temperature, and threshed, with minimal exposure to direct sunlight. One hundred kernels were drawn from seed samples for carotenoid analysis.

### Carotenoid analysis

Carotenoids were extracted from the maize kernels and quantified by HPLC at the University of Wisconsin, USA. The extraction protocol and carotenoid analysis used was the method described in [37]. Briefly, 0.5 g finely ground sample of each entry was transferred into a 50 ml glass centrifuge tube to which 6 ml of Ethanol plus 0.1% butylated hydroxyl toluene were added, vortexed for 15 seconds, and incubated in 85°C water bath for 5 min. 500 μl of 80% potassium hydroxide (w/v) was added to each sample, vortexed for 15 seconds, and incubated in the 85°C water bath for 10 min with vortexing at about 5 min interval. Samples were then immediately placed on ice and 3 ml ice cold deionized water added to each of them, vortexed for 15 seconds, and 200 μl internal standard β-Apo-8′-carotenal and 4 ml hexane added. After vortexing and centrifugation, the top hexane layer formed was transferred into a new test tube. The hexane extraction was repeated twice, adding 3 ml hexane each time. Samples were allowed to dry down completely under nitrogen gas using a Turbovap LV concentrator (Caliper Life Sciences) and reconstituted in 500 μl of 50:50 Methanol:Dichloroethane.

Fifty micro-liter aliquots of each extract were injected into an HPLC system (Water Corporation, Milford, MA). The Water’s HPLC components was operated with Empower 1 software and included a 717 Plus auto sampler with temperature control set at 5°C, Waters 1525 binary HPLC pump, and a 2996 photodiode array detector for carotenoid quantification. Carotenoids were separated by C30 Carotenoid Column (4.6 × 250 mm; 3 μm) eluted by a mobile phase gradient from 100% methanol/water (92:8 v/v) with 10 mM ammonium acetate to 50% methyl tertiary butyl ether. The flow rate was 1.0 mL/min and the solvents were HPLC grade. To maximize detection of carotenoids, absorbance was measured at 450 nm. Alpha-carotene, β-carotene (*cis* and *trans* isomers), β-cryptoxanthin, lutein, and zeaxanthin were quantified.

Total carotenoid was calculated as the sum of concentrations of α-carotene, lutein, β-carotene, β-cryptoxanthine and zeaxanthine. Provitamin A was calculated as the sum of β-carotene and half of each of β-cryptoxanthin and α-carotene concentrations, since the latter two contribute 50% of the value of β-carotene as provitamin A [38]. Other derived carotenoid traits were also calculated as indicated in [12] and [34], namely the ratio of carotenoids in β to α branch of the carotenoid pathway, the ratio of β-carotene to β-cryptoxanthine and the ratio of β-carotene to total carotenoids. The natural logarithms of the ratios were calculated to allow statistical analysis of the data, as the ratios followed a highly non-normal distribution. All concentrations were described in μg g^-1^ dry weight (DW).

### PCR based genotyping

For PCR based genotyping of the functional DNA markers, leaf samples were collected from 3 to 4 randomly selected plants of each inbred line within one of the replications of the field trial described above at 40 days after planting. DNA samples were isolated from freeze dried leaf samples of each genotype using either a CTAB (cetyl trimethyl ammonium bromide) based DNA extraction protocol or QIAGEN DNeasy^®^ Plant Mini kit (Qiagen Inc., Hilden, Germany) following the company’s protocol.

PCR based functional markers of three genes *lcy*E, *crtRB1* and *PSY1* were deployed across all the 130 inbred lines. PCR conditions, cycling profiles and primers used were based on those reported by [12] for *lcy*E [34], for *crtRB1* and [33] for *PSY1.* The primers used to amplify the *lcyE-*3′TE indel marker were forward 5′-ACCCGTACGTCGTTCATCTC-3′ and reverse 5′-ACCCTGCGTGGTCTCAAC-3′ [35]. Primer oligos were obtained from Integrated DNA Technology Inc (IDT, Belgium). All PCRs were run using BIOTAQ™ DNA polymerase kit (Bioline Ltd, UK) with a mixture composed of 2 μl 10x NH4 PCR buffer, 1 μl of each primer, 1 or 1.5 μl (depending on the marker) of 50 mM MgCl_2_, 0.15 μl of BIOTAQ™ polymerase, 1 μl of Dimethyl Sulfoxide (DMSO) to enhance specificity, and ultra pure water making up to 25 μl total volume. PCR fragments were confirmed by sequencing three samples representing each allele of the 6 functional markers. PCR product sequences were aligned with sequences of the three genes, downloaded from GenBank of NCBI or MaizeGDB, using CLC genomics workbench (CLC Bio, Denmark) sequence analysis software. Fragments in the PCR products were resolved using 2% w/v super fine resolution (SFR™) agarose gel. The names of polymorphic sites of each gene and the nature of polymorphisms are indicated in Table 2 according to their respective references.

**Table 2:** **Nomenclature of functional DNA markers and their allelic series**[12,33,34]

**Gene**	**Polymorphic site/marker gene name-polymorphism)**	**Nature of polymorphism**	Allelic series and notations*
*PSY1*[33]	*PSY -SNP7*	A-C substitution SNP	**A**, C
	*PSY1-IDI*	378 bp indel	0, **378**
*LCYE*[12]	*LCYE-5′TE*	285 indel	**1**, 2, 3, **4**
	*LCYE-SNP (216)*	G-C SNP	**G**, T
	*LCYE-3′indel*	8 bp indel	8, **0**
*crtRB1 *[34]	*crtRB1-5′TE*	397/206 bp indel	1, **2**, 3
	*crtRB1-InDel4*	12 bp indel	**12**, 0
	*crtRB1-3′TE*	325/1250 bp indel	**1**, 2, 3

*Allelic variants denoted in bold face underlined letters represent the best favourable alleles as proposed in the references. In the current study *lcyE 5′TE* yielded no amplification for 73% of the inbred lines invariably. Hence there is an additional notation in the results section for those samples scored as a  0’ allele to mean  no amplification’.

### Statistical analysis

The carotenoid data was analyzed using PROC MIXED procedure of SAS version 9.3 (SAS Institute, Cary NC) based on alpha lattice design in which lines were treated as fixed effects, while blocks, replications, years, and year by line interaction were treated as random effects. Estimates of repeatability were calculated as indicated in [33]. Letters for mean separation were generated using a SAS macro [39]. Spearman rank correlation coefficient was calculated using SAS 9.3 (SAS Institute, Cary NC) to test the consistency of ranking of the inbred lines for accumulation of carotenoids across seasons [13].

Associations between variation in carotenoid concentration and markers of each gene were calculated using the mixed linear model (MLM) [40] implemented in TASSEL version 3.0 [41]. MLM incorporates population structure and kinship in the analysis to control spurious association results [40]. Best linear unbiased estimates (BLUEs) calculated via the generalized linear model (GLM) option by selecting only the phenotype data was used for association analysis combined across the two seasons [41]. Linkage disequilibrium between functional markers was also calculated using the same software. Population structure (principal component analysis, PCA) and kinship of the 130 inbred lines were estimated within TASSEL 3.0 using 62,000 SNPs that covered the 10 maize chromosomes generated by genotyping by sequencing (GBS) method at the Institute for Genomic Diversity (IGD), Cornell, USA, according to [42]. The SNPs were filtered out from the GBS pipeline output using a threshold of 5% minimum allele frequency and 20% maximum missing data. In addition, 2328 SNPs were filtered using 0% missing and 5% minimum minor allele frequency and used for hierarchical clustering of SNP data for 26 inbred lines that harbored the favourable alleles of the two most significantly associated markers (*crtRB1-5′TE* and -*3′TE*) to assess their genetic diversity. The unweighted pair-group method with arithmetical averages (UPGMA) provided in PowerMarker version 3.25 [43] was employed to construct a dendrogram from Nei’s 1972 frequency based distance matrix [44]. Single and two-way ANOVA were conducted to determine genotype effects of the functional polymorphisms using PROC GLM and PROC MIXED of SAS 9.3 (SAS Institute, Cary NC).

## Results

### Carotenoid profiles and levels are diverse across the maize inbred lines

**Figure 1:**
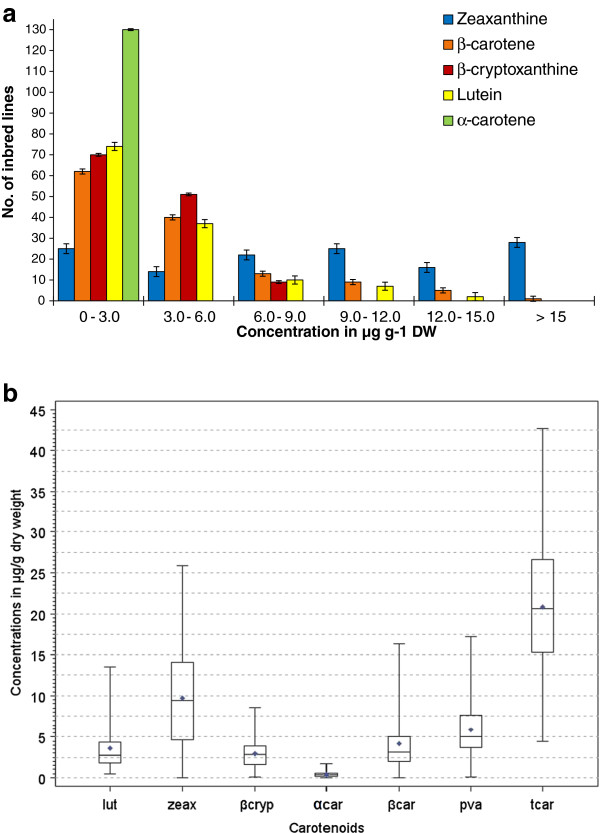
**Distribution of mean concentrations of carotenoids for 130 inbred lines. a)** Histogram; error bars represent standard error of least square means of the respective carotenoid concentration. **b)** Box plots; endpoints of upper and lower whiskers represent maximum and minimum concentrations, respectively; upper and lower edges of boxes represent third and first quartiles, respectively; line inside box represent median; symbol ♦, represent mean. Abbreviations of carotenoids described under Table 3.

The analysis of variance (ANOVA) combined over the two years revealed highly significant variation among the maize inbred lines for all carotenoids, except for α-carotene (Table 3). The effects of year, and year by line, interaction were significant on all carotenoids, except β-cryptoxanthine. High repeatability estimates ranging from 62 to 89%, were recorded for all carotenoids expect for α-carotene demonstrating the importance of the genetic component of the total variation observed for the traits. Replication did not have a significant effect on all carotenoids. Spearman’s rank correlation coefficients across years were significant (p < 0.001) for β-carotene (r = 0.83), β-cryptoxanthine, (r = 0.75) zeaxanthine (r = 0.86) α-carotene (r = 0.30) and lutein (r = 0.49). Zeaxanthine was the dominant carotenoid identified with average mean value of 9.66 μg g^-1^ followed by β-carotene, 4.21 μg g^-1^ and lutein, 3.58 μg g^-1^. The α-carotene contents of most of the maize inbred lines were very low and not significantly different from zero (apart from16 inbred lines). Estimated means averaged over the two years varied from 0.45 to 13.51 μg g^-1^ for lutein, from 0.04 to 25.90 μg g^-1^ for zeaxanthine, from 0.08 to 8.55 μg g^-1^ for β-cryptoxanthine, 0 to 16.38 μg g^-1^ for β-carotene, from 0 to 17.25 μg g^-1^ for provitamin A, and from 4.43 to 42.71 μg g^-1^ for total carotenoids (Table 4, Figure 1a and b).

**Table 3:** Combined ANOVA for carotenoid content of 130 inbred lines evaluated in 2010 and 2011

**Sources of variation**	**DF**	**Mean squares of carotenoids**^**a**^						
		**lut**	**zeax**	**βcry**	**αcar**	**βcar**	**pva**	**tcar**
**Line**	129	26.81***	133.99***	12.37***	0.43	40.71***	41.49***	226.72***
**Year**	1	519.32***	878.82**	7.95	51.98***	48.63**	84.07**	332*
**Rep (Year)**	2	2.81	22.07	0.69	0	0.57	1.29	23.58
**Block** (Rep*Year)	36	3.75**	13.43***	0.44	0.01*	0.88	1.36	31.32***
Line*Year	129	10.32***	14.21***	1.62***	0.37***	5.21***	6.63***	43.45***
**Residual**	222	2.15	4.08	0.48	0	0.76	1.01	11.28
**r**		0.62	0.89	0.87	0.15	0.87	0.84	0.81

^a^Carotenoids are abbreviated as *lut* Lutein, *zeax* Zeaxanthine, *βcry* β-cryptoxanthine, *αcar* alpha-carotene, *βcar* β-carotene, *pva* provitamin A, *tcar* Total carotenoid, *r* repeatability, *DF* degrees of freedom.

*, **, *** = significant at P ≤ 0.05, 0.01, and 0.001, respectively.

**Table 4:** **Carotenoid levels and *lcyE *** **and *crtRB1 *** **genotypes of selected maize inbred lines**

	Carotenoids* (μg g^**-1**^**dry weight)**							Genotype**					
**Line entry no.**	**lut**	**zeax**	**βcry**	**αcar**	**βcar**	**tpva**	**tcar**	***lcyE***			***crtRB1***		
								**5′TE**	**SNP (216)**	**3′ InDel**	**5′ TE**	**InDel 4**	**3′ TE**
39	1.70ab	2.72c	0.44c	1.14a	16.38a	17.25a	22.88abc	0’	**G**	8	**2**	0	**1**
45	1.03b	2.39c	0.34c	0.97a	14.79ab	15.45ab	19.53bc	0’	**G**	8	***2***	0	**1** **|**3
124	0.99b	1.12c	0.16c	1.18a	13.64abc	14.33ab	17.08bc	3	T	**0**	2	0**|** **12**	**1** **|**3
106	1.80ab	0.26c	1.30c	1.21a	11.80abc	13.02abc	16.30bc	0’	**G**	8	**2**	**12**	**1**
99	5.80ab	2.32c	0.39c	1.20a	11.30abc	12.11abc	21.10abc	3	T	**0**	**2**	0	**1**
23	5.76ab	4.83bc	0.75c	1.02a	10.77abc	11.63abc	23.04abc	0’	**G**	8	**2**	**12**	**1**
101	12.09a	5.02bc	0.83c	1.39a	9.76abcd	10.91abc	29.19abc	0’	**G**	8	**2**	**12**	**1**
107	1.90ab	1.05c	0.33c	0.80a	9.07abcd	9.65abc	13.17 c	0’	**G**	8**|** **0**	**2**	**12**	**1**
11	2.03ab	10.80abc	1.99bc	0.83a	7.61bcd	9.06abc	23.48abc	0’	**G**	8	1	0	3
50	7.28ab	18.74a	8.55a	0.57a	7.54bcd	12.10abc	42.71a	3	T	8**|** **0**	1	0	3
92	3.96ab	15.56ab	6.02ab	0.70a	6.72 cd	10.10abc	32.98abc	0’	G	8	1	**12**	3
98	3.22ab	9.43abc	2.59bc	0.77a	6.30 cd	7.98bc	22.27abc	**1**	**G**	**0**	1	0	**1** **|**3
120	1.37ab	10.36abc	1.92bc	0.60a	6.27 cd	7.52bc	20.46bc	0’	**G**	8	1	0	3
96	8.89ab	19.82a	3.28bc	0.37a	2.93d	4.76c	35.41ab	0’	**G**	**0**	1	0	3
**SE**	1.95	2.36	0.67	0.45	1.23	1.40	3.53						
**Min.**	0.45	0.04	0.08	0	0.03	0.06	4.43						
**Max.**	13.51	25.90	8.55	1.68	16.38	17.25	42.71						
**Grand mean**	3.58	9.66	2.92	0.4	4.21	5.87	20.78						

*Means within column followed by same letters are not significantly different using least square means and Tukey’s multiple comparison test (p < 0.05). Abbreviations of carotenoids are described in legend for Table 3.

**Allelic series of genotypes composed of six polymorphisms: *lcyE 5′TE, lcyE SNP (216), lcyE 3′indel, crtRB1 5′TE, crtRB1 InDel4, and crtRB1 3′TE* from left to right (discussed in later sections). Heterozygosity of the T allele of the dominant marker SNP (216) is not determined. Heterozygous alleles are separated by  |’. Favourable alleles are bolded and underlined; description of the markers is presented in Table 2. The haplotype 3, T, 8|–, 1, 0, 3 represents the worst allelic combination. The haplotype  0’, G, 8|0, 2, 12, 1 represents the best allelic combination.

### Several of IITA’s tropical adapted inbred lines harbour alleles of *lcyE* and *crtRB1* markers proposed for elevated provitamin A in maize endosperm

The two markers of *PSY1*[33] were monomorphic for the favourable allelic variants across all 130 inbred lines, and were thus not considered for further analysis. In contrast, all the PCR markers for *lcyE* and *crtRB1* were polymorphic across the inbred lines. Sequences of all sampled PCR fragments were aligned to their corresponding gene sequences (data not presented) confirming their identity. Alleles 2 and 4 of the 5′TE polymorphic site of *lcyE*, and allele 2 of the 3′TE and allele 3 of the 5′TE polymorphisms of *crtRB1*were not detected in this study (Table 5). Frequencies of the *lcyE* favourable alleles varied from 12% to 83%, while those of *crtRB1* varied from 18% to 19% (Table 5).

**Table 5:** **Observed alleles and frequencies of the favourable allelic class of *PSY1*, *lcyE *** **and *crtRB1 *** **functional markers**

**Marker**	**Expected allelic series**	Allelic variants observed**	**Favourable allele**	**Frequency of the favourable allele (%)**
*PSY1* SNP7	A, C	A	A	100
*PSY1* InDel1	0, 378	0	0	100
*lcyE* 5′TE*	1, 2, 3, 4	0’, 1, 3	1	12
*lcyE* SNP (216)	G, T	G, T	G	83
*lcyE* 3′indel	8, 0	8, 0	0	38
*crtRB1* 5′TE	1, 2, 3	1, 2	2	18
*crtRB1* indel4	12, 0	12, 0	12	19
*crtRB1* 3′TE	1, 2, 3	1, 3	1	18

*For lcyE 5′TE the vast majority of the inbred lines (> 70%) did not yield any amplification, and thus scored as  0’ alleles to represent no amplification, not deletion.

**There were individuals that were heterozygous for some of the marker classes. Description of markers and expected alleles are presented in Table 2.

The favourable alleles identified by the *crtRB1*-*5′TE* and *crtRB1*-*3′TE* markers were present in 26 inbred lines, co-occurring in 20 inbred lines. The two polymorphisms showed high linkage disequilibrium (R^2^ = 0.76). However, both (i.e. *crtRB1*-*5′TE* and *crtRB1*-*3′TE* markers) were not in linkage disequilibrium with the *crtRB1-indel4* marker (Table 6). Linkage disequilibrium values between markers of *lcyE* and *crtRB1* genes were low with R^2^ values ranging from 0.004 to 0.188.

**Table 6:** **Linkage disequilibrium between markers of *crtRB1 *** **and *lcyE *** **within the 130 yellow maize inbred lines**

**R**^2^*	**lcyE 5′TE**	**lcyE SNP (216)**	**lcyE 3′indel**	**crtRB1 5′TE**	**crtRB1 indel4**	**crtRB1 3′TE**
lcyE 5′TE		7.29E-11	7.68E-13	1.09E-03	3.11E-01	8.50E-03
lcyE SNP (216)	0.3810		3.92E-07	1.43E-05	5.66E-01	1.82E-04
lcyE 3′indel	0.3990	0.2050		1.53E-01	1.13E-01	1.01E-01
crtRB1 5′TE	0.1010	0.1880	0.0201		1.55E-01	7.88E-19
crtRB1 indel4	0.0115	0.0041	0.0208	0.0166		2.48E-01
crtRB1 3′TE	0.0658	0.1350	0.0262	0.7570	0.0144	

*Upper triangle contains p-value and lower triangle contains R^2^.

**Figure 2:**
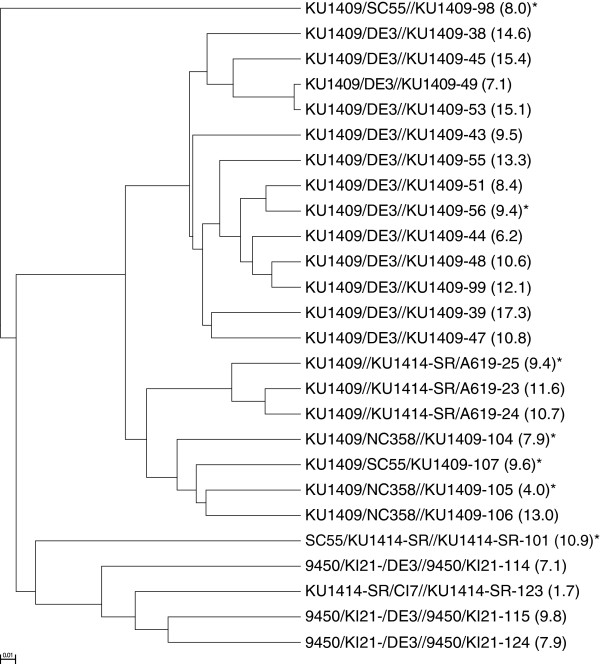
**Dendrogram of 26 inbred lines that have the best favourable alleles of crtRB1-5′TE and crtRB-3′TE marker.** The pedigrees refer to the sources from which the inbred lines were derived. The numbers after the pedigrees are inbred line entry numbers. Numbers in parenthesis are mean β-carotene concentration in μg^-1^ DW. Entry 99 is the line used in [35] for developing segregating populations. Twenty of the inbred lines contained the favourable alleles of both markers except for those marked with *.

Five different donor lines were determined to have contributed the favourable alleles of *crtRB1-5′TE* and *3′TE* to the 26 inbred lines carrying either one or both of these favourable alleles. The largest proportion of the inbred lines having favourable alleles of *crtRB1-5′TE* and/or -*3′TE* were derived from backcrosses containing a temperate inbred line DE3 as a donor parent. These inbred lines are among those lines that exhibited the highest levels of provitamin A. In addition, three tropical lines were the recurrent parents of the best inbred lines, also carrying favourable alleles. Cluster analysis based on Nei’s 1972 frequency based distance using UPGMA separated the 26 best “favourable-allele-carrying” inbred lines into three major groups with one line separated from the major groups (Figure 2). Even though lines originating from the same backcross were grouped together; they showed considerable levels of within group diversity.

### *crtRB1* functional markers had the largest effect on provitamin A variation across the 130 inbred lines

**Figure 3:**
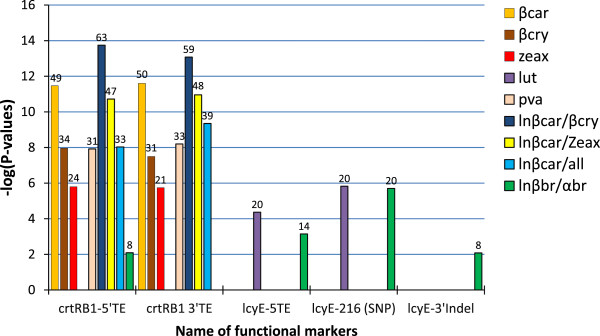
**Significant marker-trait associations estimated using BLUEs calculated based on the two year field data.** Significance thresholds: –log (1.7E-04) = 4 at α = 0.01 and –log (8.3E-3) = 2 at α = 0.05. Carotenoid names abbreviations described in Tables 3 and 7. R^2^ Values indicated on top of each bar represent percent variation explained.

Association analyses were conducted to determine the relationship between polymorphic alleles (for *lcyE and crtRB1)* and phenotypes (carotenoid levels and profiles) based on each year mean and means averaged over two years. Alpha-carotene was excluded as a phenotype from the analysis due to its extremely low concentrations and lack of significant variability among the maize inbred lines. The results of the association analysis are presented in Table 7 and Figure 3. The 3′TE and 5′TE polymorphic sites of the *crtRB1* candidate gene were found to be significantly associated with carotenoids, and all the derived traits, consistently over the two years (α = 0.01). The exceptions were lutein levels which were not affected by both *crtRB1* markers, and the α to β branch carotenoids ratio which was not affected by *crtRB1*-5′TE in the second year. The two *crtRB1* markers explained from 13 to 53% of the variation in carotenoids and derived traits in the first year, and from 17 to 63% in the second year inferring from the R^2^ values (Table 7). *CrtRB1-*indel4 accounted for 9% of the variation in provitamin A (α = 0.5). The functional DNA markers for *lcyE*, though not consistent, were significantly associated only with lutein, and the ratio of β to α branch carotenoids, explaining 15 to 21% of the variations. However, *lcyE*-5′TE did not significantly affect β to α branch ratio in the first year and *lcyE*-3′indel was not associated with any of the traits. None of the markers for each gene had significant effects on total carotenoid content in both years which is consistent with previous association analyses results [12,34]. However, due to variation in results of association and segregation mapping, earlier studies detected significant reduction of total carotenoids for genotypes with favourable alleles of *lcyE* and *crtRB1* using segregating populations [34,35].

**Table 7:** **Marker-trait association of *crtRB1 *** **and *lcyE *** **with carotenoid content of 130 yellow maize inbred lines**

		Functional DNA (PCR) markers**											
**Season**	Carotenoids*	***crtRB1 *** **5′TE**		***crtRB1 *** **Indel4**		***crtRB1 *** **3′TE**		***lcyE *** **5TE**		***lcyE *** **216(SNP)**		***lcyE *** **3′Indel**	
		**P**	**R**^**2**^	**P**	**R**^**2**^	**P**	**R**^**2**^	**P**	**R**^**2**^	**P**	**R**^**2**^	**P**	**R**^**2**^
Year 1	βcar	6.6E-13	0.53			1.2E-12	0.51						
	βcry	2.8E-08	0.32			1.5E-07	0.28						
	zeax	3.7E-08	0.31			1.5E-07	0.28						
	lut							3.1E-03	0.11	1.6E-03	0.08		
	pva	1.2E-07	0.26	2.3E-03	0.09	6.5E-07	0.23						
	lnβcar/βcry	8.6E-12	0.47			7.7E-11	0.42						
	lnβcar/Zeax	4.1E-11	0.45			1.7E-10	0.42						
	lnβcar/all	5.4E-08	0.30			3.7E-08	0.30						
	lnβbr/αbr	1.3E-04	0.15			4.7E-04	0.13			6.7E-04	0.10		
Year 2	βcar	4.4E-10	0.38			7.9E-11	0.42						
	βcry	4.6E-08	0.30			7.7E-08	0.29						
	zeax	1.2E-05	0.20			9.5E-06	0.20						
	lut							1.2E-04	0.18	2.7E-05	0.15	6.9E-04	0.12
	tpva	2.2E-05	0.17			4.8E-06	0.20						
	lnβcar/βcry	2.1E-14	0.63			1.3E-13	0.58						
	lnβcar/Zeax	8.1E-11	0.44			1.3E-11	0.47						
	lnβcar/all	5.4E-09	0.34			2.5E-10	0.41						
	lnβbr/αbr							2.6E-04	0.16	9.1E-07	0.21	5.8E-05	0.17
Combined***	βcar	3.4E-12	0.49			2.5E-12	0.50						
	βcry	1.1E-08	0.34			3.2E-08	0.31						
	zeax	1.6E-06	0.24			1.8E-06	0.23						
	lut							4.3E-05	0.20	1.5E-06	0.20		
	tpva	1.2E-08	0.31			6.3E-09	0.33						
	lnβcar/βcry	1.8E-14	0.63			8.4E-14	0.59						
	lnβcar/Zeax	1.9E-11	0.47			1.1E-11	0.48						
	lnβcar/all	9.3E-09	0.33			4.5E-10	0.39						
	lnβbr/αbr	8.1E-03	0.08					7.2E-04	0.14	2.0E-06	0.20	8.3E-03	0.08

*Carotenoid abbreviations are described under Table 3. βbr/αbr = β-branch/α-branch carotenoids,

βcar/βcry = β-carotene/β-cryptoxanthine, βcar/Zeax = β-carotene/zeaxanthine; βcar/all = β-carotene/total carotenoids.

** Only significant values are indicated. Names of functional markers for each gene are described in Table 2. Significance thresholds are 1.67E-04 and 8.3E-3 at 1% and 5%, respectively, after Bonferroni multiple test correction (alpha/number of markers analyzed per trait, i. e., 0.01/6 = 1.7E-04 and 0.05/6 = 8.3E-3) (TASSEL user’s guide 2011).

***BLUEs calculated from the two year data illustrated using bar graph in Figure 3.

### Combinatorial effects of *lycE* and *crtRBI* functional markers on carotenoid levels and profiles

Fourteen unique genotypes were observed for *lcyE* and eight unique genotypes for *crtRB1* (Table 8). The two-way ANOVA combining the *lcyE* and *crtRB1* alleles revealed highly significant interaction effects for each carotenoid type and the derived traits. The combined effects of the alleles of the two genes were stronger than their separate effects. The two genes model explained 38 to 89% of the total variation in carotenoid concentration. Individual effects of the alleles were also highly significant for almost all carotenoids. The combined *lcyE* markers explained the least variation in the β-branch carotenoids (β-cryptoxanthine, β-carotene, zeaxanthine), while the*crtRB1*markers explained the least variation in the α-branch carotenoid (lutein). The combined *crtRB1* markers had larger effects on individual and total provitamin A carotenoids in comparison to the effects of the *lcyE* markers.

**Table 8:** **Phenotypic variation explained (R**^**2**^**) by individual and combined effects of *lcyE *** **and *crtRB1 *** **alleles**

Genotype combination*	**No. of loci per genotype**	**No. of unique genotypes**	**R**^2^**									
			**lut**	**zeax**	**βcry**	**βcar**	**pva**	**tcar**	**βbr/αbr**	**βcar/βcry**	**βcar/zeax**	**βcar/all**
**1.***lcyE:5′TE, SNP (216), 3′TE; +crtRB1: 5′TE, Indel4, 3′TE*	6	34	0.68	0.42	0.44	0.83	0.71	0.38	0.52	0.89	0.77	0.77
**2.***lcyE: 5′TE, SNP (216), 3′TE*	3	14	0.42	0.22	NS	0.24	0.23	0.24	0.30	0.23	0.22	0.20
**3.***crtRB1: 5′TE, Indel4, 3′TE*	3	10	0.24	0.35	0.28	0.63	0.48	NS	0.23	0.71	0.60	0.50

*Names and symbols of polymorphic sites and their allelic variants are described in Table 2. **R^2 ^from combined ANOVA computed for each of the 3 haplotype scenarios across 2 years. NS, non-significant at alpha 0.5, the rest are significant at alpha < 0.0001. Abbreviation of carotenoids’ names described under Table 2 and 3.

Analysis of combinations of all of the six markers identified 34 unique genotypes (Table 9). The vast majority of these genotypes were represented by only one inbred line each. The most common genotype,  0’, G, 8|-, 1, 0, -|3 (corresponding to *lcyE-*5′TE, -SNP (216), -3′InDel, *crtRB1-5′TE, -indel4,* 3′TE; where the symbol '|' separates the two alleles of heterozygous loci, while the symbol  -’ represents any of the alternative alleles for the particular locus) was present in 49% of the inbred lines. The average estimated effect of the most frequent genotypes on beta-carotene was 3.54 μg g^-1^, (which was 2.45 μg g^-1^ less than the average effect of all the genotypes and 6.9 μg g^-1^ less than the average effect of those genotypes containing the favourable alleles of both *crtRB1* 5′TE and 3′TE) (Table 9). Genotype,  0’, G, 8|0, 2, 12, 1 contained the most optimal allelic combinations as it carried favourable alleles for 5 of the 6 loci and was present only in one inbred line (Entry 107) derived from a backcross KU1409/SC55/KU1409 (Table 1, Table 4). Its estimated effects were 9.05 μg g^-1^ for β-carotene, 0.33 μg g^-1^ for β-cryptoxanthine, 0.96 μg g^-1^ for zeaxanthine and 13.29 μg g^-1^ for total carotenoid. Although this genotype was predicted to be the best in terms of its allelic composition for the 6 markers, seven other genotypes were found to be superior to this genotype in their estimated levels of β-carotene. The presence of an unfavourable *lcyE* insertion in the homozygous state did not alter the effect of this genotype significantly, based on the observation of the effects on carotenoids observed in another genotype  0’, G, 8, 2, 12, 1 (N = 4), which lacked the *lcyE-*3′ insertion. Genotype  0’, G, 8, 2, 0, 1|- (N = 3) showed significantly better effect (p < 0.01) than the genotype with the predicted best allelic composition ( 0’, G, 8|0, 2, 12, 1), and had the strongest positive effect with an estimated average concentration of 15.03 μg g^-1^ for β-carotene and 15.08 μg g^-1^ for provitamin A. The major difference between the two genotypes is the lack of the favourable 12 bp insertion at *crtRB1-*indel4 in the former, which shows the negligible effect of this marker as was observed in the association analysis. Three inbred lines derived from KU1409/DE3/KU1409 contained the genotype  0’, G, 8, 2, 0, 1|-.

**Table 9:** **Observed maize line genotypes, allele frequencies and estimated average effects of combined markers of *lcyE *** **and *crtRB1 *** **on carotenoid content of 130 yellow maize inbred lines**

	**lcyE + crtRB1**						**N**	**Carotenoids ± SE**						
**Serial. no.**	**5′TE**	**SNP (216)**	**3′ InDel**	**5′TE**	**Indel4**	**3′TE**		**βcar**	**PVA**	**tcar**	**lnβbr/αbr**	**lnβcar/βcry**	**lnβcar/zeax**	**lnβcar/all**
1	** 0’**	**G**	**8**	**2**	**0**	**1**	2	15.28 ± 0.75	16.18 ± 0.95	19.83 ± 2.86	2.11 ± 0.3	3.46 ± 0.26	2.39 ± 0.39	1.52 ± 0.35
2	** 0’**	**G**	**8**	**2**	**0**	**1|3**	1	14.78 ± 1.03	15.42 ± 1.28	19.15 ± 3.99	2.15 ± 0.4	3.88 ± 0.36	2.02 ± 0.52	1.25 ± 0.48
3	**3**	**T**	**0**	**2**	**12|0**	**1|3**	1	13.65 ± 1.03	14.31 ± 1.28	18.24 ± 3.99	2.03 ± 0.4	4.34 ± 0.36	2.39 ± 0.52	1.56 ± 0.48
4	**3**	**T**	**8**	**2**	**0**	**1**	1	12.26 ± 1.03	13.16 ± 1.28	20.09 ± 3.99	1.69 ± 0.39	3.08 ± 0.36	1.27 ± 0.52	0.52 ± 0.48
5	**3**	**T**	**8|0**	**2**	**0**	**1**	2	11.75 ± 0.75	12.06 ± 0.95	17.82 ± 2.86	1.26 ± 0.3	3.74 ± 0.26	2.38 ± 0.39	0.96 ± 0.35
6	** 0’**	**G**	**8**	**2**	**12**	**1**	4	10.55 ± 0.57	11.6 ± 0.72	22.38 ± 2.08	0.88 ± 0.24	2.64 ± 0.19	1.49 ± 0.31	0.03 ± 0.26
7	**1**	**G**	**0**	**2**	**0**	**1**	1	9.23 ± 1.03	9.85 ± 1.28	11.75 ± 3.99	2.05 ± 0.39	3.23 ± 0.36	1.77 ± 0.52	1.05 ± 0.48
8	** 0’**	**G**	**8|0**	**2**	**12**	**1**	1	9.05 ± 1.03	9.61 ± 1.28	13.29 ± 3.99	1.28 ± 0.4	3.63 ± 0.36	2.53 ± 0.52	0.92 ± 0.48
9	**3**	**T**	**0**	**2**	**0**	**1**	4	8.98 ± 0.57	9.91 ± 0.72	22.56 ± 2.09	0.38 ± 0.24	2.48 ± 0.19	1.2 ± 0.31	-0.38 ± 0.26
10	** 0’**	**G**	**8**	**2**	**12**	**3**	1	8.69 ± 1.03	9.53 ± 1.28	21.20 ± 3.99	0.75 ± 0.4	2.47 ± 0.36	0.52 ± 0.52	-0.49 ± 0.48
11	**3**	**T**	**8|0**	**1**	**0**	**3**	1	7.56 ± 1.03	12.09 ± 1.28	43.36 ± 3.99	1.72 ± 0.39	-0.11 ± 0.36	-0.9 ± 0.52	-1.51 ± 0.48
12	**3**	**T**	**8|0**	**2**	**0**	**1|3**	1	7.11 ± 1.03	8.31 ± 1.28	28.58 ± 3.99	0.08 ± 0.4	1.53 ± 0.36	0.17 ± 0.52	-1.12 ± 0.48
13	**1|3**	**T**	**0**	**2**	**0**	**1**	1	6.63 ± 1.03	7.10 ± 1.28	13.09 ± 3.99	0.77 ± 0.4	2.58 ± 0.36	1.51 ± 0.52	0.05 ± 0.48
14	** 0’**	**G**	**8**	**1**	**0**	**1|3**	1	6.47 ± 1.03	7.90 ± 1.28	23.05 ± 3.99	2.94 ± 0.39	0.55 ± 0.36	-0.73 ± 0.52	-0.95 ± 0.48
15	**1**	**G**	**0**	**1**	**0**	**1|3**	1	6.33 ± 1.03	8.00 ± 10.28	22.46 ± 3.99	1.60 ± 0.4	0.92 ± 0.36	-0.47 ± 0.52	-0.98 ± 0.48
16	**3**	**T**	**0**	**1**	**0**	**3**	1	6.09 ± 1.03	9.38 ± 1.28	34.21 ± 3.99	1.38 ± 0.4	0.04 ± 0.36	-0.75 ± 0.52	-1.47 ± 0.48
17	** 0’**	**T**	**8**	**2**	**0**	**1**	1	6.01 ± 1.03	7.04 ± 1.28	10.35 ± 3.99	1.66 ± 0.4	1.59 ± 0.36	1.57 ± 0.52	0.37 ± 0.48
18	** 0’**	**G**	**8**	**1**	**12**	**3**	6	3.96 ± 0.5	5.80 ± 0.63	27.06 ± 1.75	1.62 ± 0.22	0.49 ± 0.16	-0.77 ± 0.28	-1.19 ± 0.22
19	**1**	**G**	**0**	**1**	**0**	**3**	2	3.83 ± 0.75	5.52 ± 0.95	16.87 ± 2.86	1.39 ± 0.3	0.21 ± 0.26	-0.45 ± 0.39	-1.29 ± 0.35
20	**1|3**	**T**	**0**	**1**	**12**	**3**	1	3.49 ± 1.03	6.12 ± 1.28	31.77 ± 3.99	1.39 ± 0.4	-0.36 ± 0.36	-1.59 ± 0.52	-2.07 ± 0.48
21	**3**	**T**	**0**	**1**	**0**	**3**	6	3.14 ± 0.5	4.83 ± 0.63	27.80 ± 1.76	1.01 ± 0.22	0.38 ± 0.16	-1.45 ± 0.28	-2.12 ± 0.22
22	** 0’**	**T**	**0**	**1**	**0**	**3**	1	3.14 ± 1.03	5.07 ± 1.28	28.38 ± 3.99	1.21 ± 0.4	-0.1 ± 0.36	-1.61 ± 0.52	-2.12 ± 0.48
23	** 0’**	**G**	**8**	**1**	**0**	**3**	58	2.99 ± 0.32	4.83 ± 0.42	19.91 ± 0.89	1.93 ± 0.17	-0.14 ± 0.08	-1.21 ± 0.21	-1.78 ± 0.13
24	** 0’**	**G**	**8|0**	**1**	**0**	**1|3**	1	2.85 ± 1.03	3.99 ± 1.28	6.41 ± 3.99	1.87 ± 0.4	0.42 ± 0.36	1.24 ± 0.52	-0.22 ± 0.48
25	** 0’**	**G**	**0**	**1**	**0**	**3**	9	2.84 ± 0.44	4.64 ± 0.56	24.59 ± 1.49	1.46 ± 0.2	-0.1 ± 0.14	-1.39 ± 0.26	-2.1 ± 0.19
26	**1**	**G**	**0**	**1**	**12**	**3**	7	2.70 ± 0.47	4.97 ± 0.6	21.46 ± 1.66	1.85 ± 0.21	-0.45 ± 0.15	-1.39 ± 0.27	-1.94 ± 0.21
27	** 0’**	**G**	**8**	**NA**	**0**	**3**	1	2.54 ± 1.03	4.25 ± 1.28	22.04 ± 3.99	1.66 ± 0.4	-0.21 ± 0.36	-1.54 ± 0.52	-2.04 ± 0.48
28	**3**	**G**	**0**	**1**	**0**	**3**	1	2.45 ± 1.03	4.02 ± 1.28	26.78 ± 3.99	0.28 ± 0.4	-0.06 ± 0.36	-1.37 ± 0.52	-2.4 ± 0.48
29	**1**	**G**	**8**	**1**	**0**	**3**	2	1.97 ± 0.76	3.17 ± 0.95	8.31 ± 2.88	1.44 ± 0.3	-0.13 ± 0.26	0.04 ± 0.39	-1.12 ± 0.35
30	** 0’**	**G**	**8|0**	**1**	**0**	**3**	4	1.84 ± 0.57	3.32 ± 0.72	15.86 ± 2.09	1.34 ± 0.24	-0.47 ± 0.19	-1.54 ± 0.31	-2.23 ± 0.26
31	**NA**	**G**	**0**	**NA**	**0**	**3**	1	1.82 ± 1.03	3.75 ± 1.28	22.50 ± 3.99	0.68 ± 0.4	-0.67 ± 0.36	-1.68 ± 0.52	-2.43 ± 0.48
32	** 0’**	**T**	**8**	**1**	**12**	**3**	1	1.72 ± 1.03	3.62 ± 1.28	15.98 ± 3.99	1.04 ± 0.39	-0.72 ± 0.36	-1.09 ± 0.52	-2.07 ± 0.48
33	**3**	**G**	**8**	**1|2**	**0**	**3**	1	1.05 ± 1.03	1.65 ± 1.28	13.43 ± 3.99	1.74 ± 0.4	0.03 ± 0.36	-2.12 ± 0.52	-2.42 ± 0.48
34	** 0’**	**G**	**0**	**1**	**12**	**3**	3	0.93 ± 0.64	1.71 ± 0.80	10.18 ± 2.37	1.56 ± 0.26	-0.42 ± 0.22	-1.34 ± 0.34	-1.45 ± 0.29

N = number of inbred lines, NA = Not available (missing marker score). SE = Standard error of least square means. Symbol  |’ separates heterozygous alleles of a locus. SNP (216) is a dominant marker for the  T’ allele, thus  T’ can be  TT’ or  TG’.

The favourable alleles of 5′ and 3′TE markers of crtRB1 (alleles 2 and 1) were present in almost all genotypic combinations that had large positive effects on β-carotene concentration ranging from 6.0 to 15.28 μg g^-1^. The genotype 3, T, 8|0, 1, 0, 3 did not have any of the favourable alleles except the deletion allele representing *lcyE*-3′TE. Only one inbred line (Entry number 50) derived from a backcross KU1409/DE3/KU1409 carrying this genotype had estimated average effects of 7.56 μg g^-1^ for β-carotene, 8.55 μg g^-1^ for beta-cryptoxanthine, 12.09 μg g^-1^ for provitamin A and 43.3 μgg^-1^ for total carotenoid. The total carotenoid concentration of this genotype exceeded that of the average total carotenoid of those genotypes carrying the favourable alleles of *crtRB1 5′TE* and *3′TE* by 23.48 μg g^-1^. This genotype also had 7.32 μg g^-1^ higher β-cryptoxanthine than the average of those carrying the above mentioned allelic classes. These results were corroborated by the low ratio values of beta-carotene to β-cryptoxanthine, β-carotene to zeaxanthine and β-carotene to all carotenoids. Another exceptional genotype 3, G, 8, 1|2, 0, 3 containing the favourable allele of crtRB1 5′TE in the heterozygous state showed a very weak effect on beta-carotene (1.05 μg g^-1^) and provitamin A (1.65 μg g^-1^) content. The weakest effect was detected from genotype  0’, G, 0, 1, 12, 3 (N = 3), which was devoid of the two best favourable alleles of crtRB1 5′TE and 3′TE (Table 4), which had relatively low level of total carotenoids (10.18 μg g^-1^). Overall, the average effects of the genotypes harbouring both favourable alleles of crtRB1-5′TE and -3′TE (N = 23) resulted in 7.2 μg g^-1^ increase or 3.22 fold increase in β-carotene as compared to the effects of genotypes without any of the favourable alleles (N = 103). The reduction in total carotenoid between the two sets of allelic classes was found to be negligible (from 23.5 to 18 μg g^-1^).

## Discussion

Maize is an important staple food crop for food and livelihood security in Africa. Since the 1970s, IITA has had a maize breeding program to develop tropical maize lines that are high-yielding and adapted to growing conditions across Africa. To help alleviate micronutrient deficiencies amongst the poor whose diets are highly dependent on maize, the development of tropical adapted maize lines with elevated levels of carotenoids, in particular provitamin A, is a major maize improvement goal.

To facilitate the development of high vitamin A tropical maize varieties, the carotenoid profile and content of 130 tropical adapted inbred lines within the conventional breeding program for tropical maize at IITA were analysed over two seasons under field conditions. Within these 130 inbred lines, 28 lines were found to have a provitamin A content of 8.0 to 17.25 μg g^-1^ which is higher than previously reported by [13] and is comparable to the highest provitamin A level described in [14]. This finding highlights the importance of introgressing the best favourable alleles of provitamin A from temperate germplasm into tropical adapted inbred lines. The maximum average estimated level of total carotenoids detected was 42.71 μg g^-1^, which was much lower than the 100 μg g^-1^ previously reported [11]. The identification of inbred lines with high total carotenoid levels is considered to be useful if the high influx of substrates to the carotenoid biosynthesis pathway favors an increase in provitamin A carotenoids in maize endosperm [32].

DNA markers detecting polymorphisms in genes that are functionally responsible for changes in phenotypes can be called functional markers [27,45]. Eight functional markers of three key carotenoid genes *PSY1*[12], *lcyE*[33] and *crtRB1*[34], (previously developed using different association panels of diverse temperate, sub-tropical and/or tropical yellow maize inbred lines), were considered for validation in this study. The functional markers for the two genes *lcyE* and *crtRB1* have been described as one of the most exciting discoveries for maize endosperm provitamin A improvement endeavors [14]. However, independent studies demonstrated some inconsistencies in the effects of these markers [11,35,36] which necessitates additional investigations so that such markers can be deployed in maize breeding in a more robust and predictable manner. In the earlier studies, the panels and populations used for developing and validating the functional markers were largely of temperate origin and had low frequencies of the favourable alleles of the most significant markers. In our field study over two years, these functional markers have been analysed for their efficacy in diverse maize inbred lines derived mainly from populations containing a mixture of tropical and temperate germplasm in their pedigrees. Our study clearly demonstrates that the effects are heritable thereby can facilitate the development of robust maize varieties with elevated provitamin A levels.

The functional markers for *PSY1* were monomorphic for the favourable allelic variants across all the inbred lines possibly because of the highly conserved nature of the *PSY1* gene within and across species [46]. For instance, Fu et al. [33] have observed that the favourable alleles of *PSY1* were fixed within the tropical genetic background of the panels used in their study. The variation in total carotenoid content observed in our study could be due either to the presence of some rare functional variation within the *PSY1* gene and its regulatory regions (of a genetic or epigenetic nature) and/or other “modifier” genes that are involved in the carotenoid biosynthesis in the genotypes tested [32].

The markers for *lcyE* and *crtRB1* were polymorphic across the maize inbred lines, where in the 3′ and 5′TE markers of *crtRB1* exhibited strong association with variation in β-carotene content of the inbred lines. In contrast, the effects of *lcyE* markers were found to be weak and inconsistent in the present study, which was in line with previous results [11,35].The germplasm within the tropical maize gene pool is known to be more diverse than that in the temperate maize gene pool. In a previous study Yan et al. [34] detected the favourable allele of *crtRB1-5′TE* only in the temperate yellow maize germplasm, with a frequency of less than 3%. In our study on tropical maize germplasm, this allele occurred at a relatively high frequency of 18%. The high linkage disequilibrium (R^2^ = 0.76) between the 3′TE and 5′TE polymorphisms of *crtRB1* detected in our study deviates from a previous report [34] that found no linkage disequilibrium (R^2^ = 0.02). It is probable that the two favourable alleles were introgressed (from temperate donor inbred lines into the tropical adapted materials) together as genetically linked alleles that led to the observed strong linkage disequilibrium between the two markers. Such linkage disequilibrium makes the estimation of the independent effect of each marker difficult. However, based on results of previous studies [34,35], it can be argued that both markers could be contributing to the strong association of *crtRB1* with provitamin A in maize endosperm, with the 5′TE polymorphism contributing the largest effect.

One of the maize lines (derived from a back cross involving the maize line DE as a donor parent) was also used for developing five different segregating populations used by [35] to test the effect of *crtRB1-3′TE* polymorphism [35]. In our study, this line along with 25 other inbred lines carried the best favourable alleles of *crtRB1-*5′TE and -3′TE polymorphisms and was used to evaluate to what extent the lines carrying the two favourable alleles in this study were different from the segregating population used in [35]. UPGMA cluster analysis of SNPs based on Nei’s 1972 distance (Figure 2) separated the lines according to their genetic backgrounds. Substantial genetic variation was found among the lines originating from the same backcross. The line used by [35] was clustered with one of the major groups thus underpinning the diversity of the lines used in our study. In previous analyses, the effect of *crtRB1-5′TE*, the marker that was reported to have the largest effect in the work of Yan et al. [34], was not reported in the validation study of [35]. Hence, our study fills a major gap by now providing the marker-trait association results for *crtRB1-5′TE* in tropical adapted maize germplasm.

Almost all of the inbred maize lines with high levels of β-carotene and total provitamin A carried the favourable alleles of the most significant functional markers of *crtRB1-*3 T’ and *crtRB1*-5′TE. An inconsistency detected in our marker-trait study was a maize inbred line that showed unexpectedly low β-carotene and provitamin A (<2.0 μg g^-1^ DW) although it carried the favourable allele of *crtRB1-*5′TE polymorphism. This inbred line also had relatively lower total carotenoid content (14.14 μg g^-1^ DW), possibly suggesting that high influx of substrates into the carotenoid biosynthesis pathway may be an important factor to realize the desired action of the favourable alleles [11,33,34]. Hence, introgression of these favourable alleles into adapted maize germplasm with high total carotenoid content could be a strategy for increasing levels of β-carotene and total provitamin A.

The combinatorial analysis of the functional polymorphisms for the two genes revealed larger effects than those observed for alleles of each gene independently. This finding is in agreement with results of previous studies [34,35]. In particular, our analysis identified a number of superior genotypes of maize inbred lines that have carotenoid levels of relevance to provitamin A level enhancement that exceed previously obtained levels within IITA’s breeding program. Our results indicates that the functional markers for *crtRB1* markers have the strongest potential to accelerate genetic gain for enhanced β-carotene content in tropical maize breeding programs [35]. Given that the carotenoid biosynthesis pathway is most likely conditioned by a number of genes and regulatory elements, and that all of the variation in provitamin A levels are not accounted for, it can be worthwhile to consider genomic selection approaches for enhanced provitamin A carotenoid levels.

## Conclusions

The first generation of provitamin A enriched maize hybrids have been developed for Nigeria and Zambia recently, which will most likely spread to other African countries with similar agro-ecologies over the coming years [47,48]. In this two year field study on tropical maize germplasm we have demonstrated the strong association between favourable alleles detected by the *crtRB1-*5′TE and -3′TE functional markers and high levels of β-carotene. Our study found these two markers to be in strong linkage disequilibrium in the tropical maize germplasm, raising the possibility that one of the polymorphic sites (e.g. the 5′TE marker) could be targeted to reduce costs associated with PCR genotyping. The high provitamin A inbred lines harbouring combinations of the favourable alleles of the *crtRB1-5’TE* and *crtRB1-3’TE* markers can be used to speed up the development of the next generation of high provitamin A tropical maize hybrids for production in Sub-Saharan Africa, thus contributing to the alleviation of hidden hunger due to vitamin A deficiency.

## Competing interests

The authors declare that they have no competing interests.

## Authors’ contributions

GA conducted the field and laboratory experiments, carried out the statistical analysis, and prepared the draft of the manuscript. AM contributed to the conception of the project, supplied maize inbred lines developed by AM, supervised the field work and revised the manuscript. MG closely supervised the laboratory work in IITA and revised the manuscript. CS contributed to the conception of the project, securing of funding, participated in the supervision of the overall work and contributed to the drafting and finalization of the manuscript. CS, AM and MG participated in the PhD supervision of GA. All authors read and approved the final manuscript.
